# IgG4 antibodies from patients with asymptomatic bancroftian filariasis inhibit the binding of IgG1 and IgG2 to C1q in a Fc-Fc-dependent mechanism

**DOI:** 10.1007/s00436-019-06451-2

**Published:** 2019-09-04

**Authors:** Ulrich F. Prodjinotho, Achim Hoerauf, Tomabu Adjobimey

**Affiliations:** 1 Institute for Medical Microbiology, Immunology and Hygiene (IMMIH), Technical University Munich, Munich, Germany; 2 Institute of Medical Microbiology, Immunology and Parasitology (IMMIP), University Hospital Bonn, Sigmund-Freud-Straße 25, 53105 Bonn, Germany; 3 Bonn-Cologne Site, German Center for Infectious Disease Research (DZIF), Bonn, Germany; 4 Département de Biochimie, Faculté des Sciences et Techniques (FAST), Université d’Abomey-Calavi (UAC), Abomey-Calavi, Bénin

**Keywords:** Lymphatic filariasis, Immune regulatory response, Complement, C1q, IgG4, Fc-Fc interactions

## Abstract

A striking feature of lymphatic filariasis (LF) is the clinical heterogeneity among exposed individuals. While endemic normals (EN) remain free of infection despite constant exposure to the infective larvae, a small group of patients, generally microfilaria free (Mf-) develops severe pathology (CP) such as lymphedema or hydrocele. Another group of infected individuals remains asymptomatic while expressing large amounts of microfilariae (Mf+). This Mf+ group is characterized by an immune-suppressed profile with high levels of anti-inflammatory cytokines and elevated IgG4. This particular immunoglobulin is unable to activate the complement. The complement system plays a critical role in both innate and adaptive immunity. However, its importance and regulation during LF is not fully understood. Using affinity chromatography and solid-phase-enzyme-immunoassays, we investigated the ability of antibody isotypes from LF clinical groups to bind C1q, the first element of the complement’s classical pathway. The results indicate that while C1q is similarly expressed in all LF clinical groups, IgG1–2 in the plasma from Mf+ individuals presented significantly lower affinity to C1q compared to EN, Mf−, and CP. In addition, selective depletion of IgG4 significantly enhanced the affinity of IgG1–2 to C1q in Mf+ individuals. Strikingly, no effect was seen on the ability of IgG3 to bind C1q in the same conditions. More interestingly, papain-generated IgG4-Fc-portions interacted with Fc portions of IgG1–2 as revealed by far-western blot analysis. These data suggest that while being unable to bind C1q, IgG4 inhibits the *first steps of the* complement classical pathway by IgG1 or IgG2 via Fc-Fc interactions.

**Electronic supplementary material:**

The online version of this article (10.1007/s00436-019-06451-2) contains supplementary material, which is available to authorized users.

## Introduction

Lymphatic filariasis (LF) is a major public health concern in tropical and subtropical countries. Despite 13 years of mass drug administration (MDA) programs, 68 million LF cases remain worldwide, of which 37 million suffer from severe pathology (lymphedema and hydrocele) (Hooper et al. 2014; Ramaiah and Ottesen 2014). In endemic areas, a variety of clinical phenotypes are present. Endemic normals (EN) are clinically asymptomatic and presented no sign of infection, while asymptomatic microfilaraemics (Mf+) present circulating microfilaria but no sign of overt disease. In contrast, chronic pathology patients (CP) present severe disease manifestations such as hydrocoele, lymphedema, or elephantiasis (Babu and Nutman 2012; Simonsen et al. 2013). These outcomes are tightly linked to the host’s immune reactivity. Typically, extracellular parasites induce Th2 immune responses. Th2-type immunity involves a cellular mobilization with an appropriate humoral response that includes secreted and excreted proteins such as cytokines, antibodies, and proteins of the complement system (Janeway et al. 2001). Alongside these classical Th2 responses, the majority of LF patients present a strong immune-regulated profile with high levels of regulatory cells, anti-inflammatory cytokines, and elevated IgG4. This particular immunoglobulin is well known to be unable to activate the complement pathway (Adjobimey and Hoerauf 2010; Prodjinotho et al. 2017). The human complement system consists of over 30 circulatory or membrane-bound plasma proteins and can be activated at the site of infection by three different pathways (classical, lectin, and alternative). These pathways converge toward the generation of C3 convertases, which proteolytically cleave C3 into C3a and C3b. C3b bound to the C3 convertases lead to the formation of C5 convertases that cleave C5 into two fragments: C5a and C5b. Finally, C5b initiates the activation of the terminal complement activation cascade (C5b-9) leading to the formation of the membrane attack complex (MAC) by covalent binding to the parasite surface and to the lysis of the parasite (Haapasalo et al. 2009; Ricklin et al. 2016). The activation of the classical pathway of the complement is initiated by complement first component C1q (Janeway et al. 2001). C1q is a 460-kDa protein assembled from three different polypeptide chains (Venkatraman Girija et al. 2013). C1q binds either directly to parasite surface components or indirectly to the Fc portion on circulating immune complexes (CIC), mostly composed of IgM or IgG subclasses (Boes et al. 1998). The binding of C1q to immune complexes and/or parasite surface and the consequent activation of C1s–C1r–C1r–C1s tetramer lead to the C3 convertase of the classical pathway (Ricklin et al. 2016), which promotes the production of effector molecules (C3a, C3b, C5a, C5b-9). Besides complement activation, C1q is also known to regulate cell differentiation, adhesion, migration, activation, and cytokine production (Elkon and Santer 2012; Lood et al. 2009; Son et al. 2012). The maturation of dendritic cells, which plays a key role in antigen processing, is increased by C1q binding (Peng et al. 2008); thus suggesting a key role of C1q in the initiation of an immune response. Interactions between T cells and dendritic cells are influenced by C1 and C3 complement fragments (Peng et al. 2008; Reis et al. 2007). These fragments were shown in filarial infections to opsonize filariae for recognition by NK cells and macrophages (Janeway et al. 2001). Recent studies demonstrated that C1q also exert immunoregulatory properties, limiting innate immune cells’ functions (Elkon and Santer 2012; Lood et al. 2009; Son et al. 2012). The fixation of immune complexes to C1q triggers a broad spectrum of pro-inflammatory effects which leads to helminth worms and/or filariae destruction. To avoid destruction by their host’s immune system, filarial parasites have developed a large range of strategies including the induction of the non-cytolytic IgG4 antibody (Adjobimey and Hoerauf 2010; Cox 2002). IgG4 is the least abundant subclass of IgG in normal human serum, representing up to 4% of all IgG (Janeway et al. 2001). IgG4 was shown in different models to exhibit immunosuppressive properties, especially via Fab-arm exchange (van der Neut et al. 2007). In contrast to IgG1, IgG2, and IgG3, IgG4 has no affinity for the complement and was even shown in some models to hinder complement activation by other antibodies (van der Zee et al. 1986). Remarkably, the Fc fragment of IgG4 was shown to interact with the Fc portion of other IgG (Rispens et al. 2009). Previous investigations in filarial infections have associated increased levels of the antibody IgG4 to the regulation of immune effector functions including complement activation (Adjobimey and Hoerauf 2010; Babu et al. 2006; Maizels and Yazdanbakhsh 2003; Rees-Roberts et al. 2010). However, the mechanism underlying complement inhibition by helminth-induced IgG4 antibody is not yet clearly established. In the present study, we demonstrated that IgG4 antibodies from filarial asymptomatic infected patients consistently reduced the ability of pro-inflammatory antibodies IgG1 and IgG2 to interact with and activate the complement C1q via Fc-Fc interactions.

## Materials and methods

### Study population, samples collection, and ethics

Patients and endemic controls’ samples were collected between 2008 and 2010 in the Nzema East District in the western region of Ghana endemic for LF. No other human filarial species were endemic in the region. Recruited individuals were part of a clinical trial (ISRCTN15216778) (http://www.filaria.eu/projects/projects/epiaf.html) (Arndts et al. 2012). Written informed consent was obtained from all participants. Persons eligible for participation were male adults in good health, 18–60 years of age, with a minimum body weight of more than 40 kg and without any clinical condition requiring chronic medication. Exclusion criteria included abnormal hepatic and renal enzyme levels (γ-glutamyltransferase > 28 U/L, glutamyl pyruvic transaminase > 30 U/L, creatinine > 1.2 mg/100 mL) assessed by dipstick chemistry, alcohol, drug abuse, or antifilarial therapy in the past 10 months. Study participants were examined by a clinician using physical methods and a portable ultrasound machine (180 Plus; SonoSite, Bothell, WA) as described previously (Debrah et al. 2009). Ethical clearance was given by the Committee on Human Research Publication and Ethics at the University of Science and Technology in Kumasi and the Ethics Committee at the University Hospital Bonn (Ethikkommission der Medizinischen Fakultät der Rheinischen Friedrich-Wilhelms-Universität Bonn). Microfilarial load was determined by microscopic examination of fingerprick night blood samples as published (Debrah et al. 2009). Subsequently, 10 mL of venous blood was collected from each eligible volunteer and plasma was taken, aliquoted, stored at − 20 °C, and then transferred to liquid nitrogen until used.

Samples include EN, residing in the endemic region but free of infection and negative for circulating filarial antigen (CFA) and microfilaraemia (Mf) (CFA−, Mf−, *n* = 22), clinically asymptomatic microfilaraemic (CFA+, Mf+, *n* = 18) and amicrofilaraemic (CFA+, Mf−, *n* = 22) subjects, positive for circulating filarial antigen and a group of chronic pathological individuals termed “CP” (*n* = 18), negative for filarial antigen.

### Preparation of plasma samples

Plasma samples from endemic normals and LF-infected individuals were collected using a ficoll gradient centrifugation method (Arndts et al. 2012; Jaatinen and Laine 2007). Ten milliliters of patient blood was poured into the ficoll containing tubes (Greiner, Frickenhausen, Germany) and centrifuged at 800×*g* for 20 min at room temperature. Thereafter, plasma samples were removed from the upper phase of the gradient, stored at − 20 °C in 1.8 ml cryotubes (Nunc, Roskilde, Denmark) and then transferred to liquid nitrogen until used.

### Determination of complement C1q levels in plasma

The level of complement first component C1q in plasma samples was determined using a specific ELISA kit from eBioscience (Vienna, Austria). Microtiter plates provided in the kit were pre-coated with an anti-human C1q antibody. After thorough washing of the wells, 100 μl of prediluted plasma samples (1:100) and prepared standards were added to their respective wells and plates were allowed to incubate for 2 h at room temperature on a microplate shaker (Grant Instruments, Cambridgeshire, UK) set at 400 rpm. Wells were then washed 6 times and each well was filled with 100 μl of prepared biotin-conjugated detection antibody and incubated for 1 h on a microplate shaker. Thereafter the plates were washed and 100 μl of streptavidin-HRP was added to the wells for 1 h with shaking in the dark. After a final washing step, wells were incubated with 100 μl of TMB substrate solution for 30 min and substrate reaction stopped with acidic stop solution. The absorbance of each microwell was read at 450 nm using the SpectraMAX ELISA reader (Molecular Devices, Sunnyvale, USA) and the concentration of C1q in samples determined.

### Depletion of IgG4 antibody

IgG4 antibodies were depleted from plasma samples of EN, Mf+, Mf−, and CP individuals using the CaptureSelect Human IgG4 affinity matrix (Life Technologies, Paisley, UK), containing an antibody fragment recognizing human IgG4, according to the manufacturer’s instructions. Briefly, CaptureSelect affinity matrix was carefully packed and equilibrated in 10 ml affinity chromatography column with PBS (pH 7.3). One hundred microliters of plasma samples was diluted with 1400 μl binding buffer (1× PBS) and loaded onto the column with a linear flow rate of 150 cm/h. Since the antibody fragment in the affinity matrix binds to IgG4 antibodies, non-IgG4 plasma components were washed out with PBS and collected as IgG4-negative plasma. Bound IgG4 was eluted in 1 ml fractions using Elution Buffer (0.1 M Glycine/HCl pH 3.0) and immediately neutralized with 1:10 volume of saturated Tris-HCl (pH 9.0). The total protein concentration in IgG4-positive and IgG4-negative plasma was then assessed at 280 nm using a NanoDrop 1000 spectrophotometer (Thermo Fisher Scientific, Wilmington, USA) and concentrations of immunoglobulin isotypes IgG1–4, IgA, IgE, and IgM were assessed by Luminex.

### Luminex immunoglobulin isotyping assay

To determine the immunoglobulin isotypes composition in IgG4-positive and IgG4-negative plasma of EN and LF patients, ProcartaPlex Human Antibody Isotyping Panels (eBioscience, Vienna, Austria) were used according to manufacturer’s instructions. Briefly, antibody-coated magnetic bead mixtures were incubated with 25 μl of assay buffer, kit standards, or diluted plasma samples (1:20000) in ProcartaPlex 96-well plates at room temperature for 1 h. Twenty-five microliters of detection antibody mixture was then added, and the plates were incubated on an orbital shaker (Stuart, Staffordshire, UK) at 500 rpm for 30 min. After that, each well was incubated with 50 μl of diluted Streptavidin-Phycoerythrin for 30 min. All incubations were performed at room temperature in the dark (plate covered with black microplate lid) and the plates washed using a hand-held magnetic plate washer. Afterward, samples were suspended in 120 μl reading buffer. Data were acquired using a MAGPIX Luminex system (Luminex Cooperation) and analyzed with ProcartaPlex Analyst software 1.0.

### Determination of IgG-C1q levels in different plasma

To measure the levels of total IgG bound to complement C1q in plasma from EN, Mf+, Mf−, and CP, an enzyme immunoassay (EIA) was used as previously described (Senbagavalli et al. 2011). Briefly, 100 μl of diluted plasma samples (1:50) was added to a C1q-precoated and rehydrated microtiter well plate (Quidel Corporation (San Diego, CA, USA)) and incubated for 1 h at room temperature. After wells had been washed 5 times, 50 μl of horseradish peroxidase-conjugated goat anti-human IgG were dispensed in each well for 30 min to detect bound IgG. An additional wash procedure was performed followed by 30 min incubation with 100 μl of substrate solution. The reaction was stopped with 50 μl of acid solution. The optical density of each well was measured at 405 nm and is proportional to the amount of IgG binding the solid-phase C1q.

### Assessment of the ability of antibody isotypes to interact with C1q

To evaluate the binding capacity of immunoglobulin isotypes in IgG4-positive and IgG4-negative plasma from EN, Mf+, Mf−, and CP individuals to C1q, high binding ELISA plates (Greiner Bio-One, Frickenhausen, Germany) were coated with 50 μl recombinant human complement C1q (Sigma-Aldrich, Saint Louis, Missouri, USA) at the concentration of 1 μg/ml and incubated at 4 °C overnight. The plates were then washed 5 times with PBS/0.05% Tween 20 and blocked with PBS/1% BSA for 1 h at room temperature. The wash step was repeated and subsequently, plates were incubated overnight at 4 °C with 50 μl/well of IgG4-positive and IgG4-negative plasma samples at various dilutions (1:1000 for IgG1–2 binding, 1:500 for IgG4, IgE, and 1:2000 for IgG3, IgM). Wells were further washed and diluted biotin-conjugated anti-IgG1, IgE (1:1000); IgG2, IgG4 (1:15000), and IgG3, IgM (1:4000) (all from Sigma Aldrich, Saint Louis, Missouri, USA) were added, followed by incubation at room temperature for 2 h. Finally, the plates were again washed and then incubated with 50 μl/well of Streptavidin-HRP for 45 min in the dark. After a final washing step, 50 μl/well TMB substrate solution were added to the plates and the reaction was stopped 15 min later with 25 μl/well 2 N H_2_SO_4_ stop solution (Merck KGAA, Darmstadt, Germany). Optical density was measured at 450 nm using the SpectraMAX ELISA reader and the results were expressed as arbitrary units (AU) relative to a unique plasma sample with the highest OD used as standard on all plates and arbitrarily set at 5 AU.

### Affinity chromatography for the purification of IgG1–2 fractions from IgG4-negative plasma

To purify IgG1–2 fractions, total IgG was first isolated from IgG4-negative plasma using prepacked HiTrap™ Protein G columns (GE Healthcare, Freiburg, Germany) according to the manufacturer’s instructions. Briefly, 100 μl of plasma samples were diluted with 1400 μl PBS and passed through a pre-equilibrated protein G-Sepharose column (GE Healthcare, Freiburg, Germany). After Protein G binds to human IgG1–3 subclasses, non-IgG plasma components were washed out from the column. Bound IgG1–3 were eluted in 1 ml fractions using IgG Elution Buffer (0.2 M Glycine/HCl, pH 3.0) and neutralized with saturated Tris-HCl (pH 9.0). Thereafter, IgG1–2 antibodies were isolated from IgG1–3 fractions of Mf+ and Mf− using prepacked Protein A columns (Thermo Fisher Scientific, Rockford, USA) following the manufacturer’s recommendations. Briefly, diluted IgG1–3 samples were passed through a pre-equilibrated protein A column. Since Protein A highly binds to all human IgG subclasses, except IgG3, non-IgG1–2 components were washed out from the column and collected. Bound IgG1–2 antibodies were eluted in 1 ml fractions using Elution Buffer (0.2 M Glycine/HCl, pH 3.0) and neutralized with saturated Tris-HCl (pH 9.0). The protein concentration was then assessed at 280 nm using a NanoDrop 1000 spectrophotometer (Thermo Fischer Scientific, Wilmington, USA).

### Preparation of the Fc fragments of IgG4 and IgG1–2 antibodies

Fc fragments of IgG4 and IgG1–2 antibodies from Mf+ and Mf− individuals were separately generated by papain digestion using Pierce Fab preparation kit (Thermo Fisher Scientific, Rockford, USA) following the manufacturer’s instructions. Digested IgG4 and IgG1–2 antibodies were further separated by centrifugation from immobilized papain. Thereafter, purification of the Fc fragments was accomplished using a Protein A affinity chromatography. Briefly, the digest was passed through Protein A spin column provided in the kit. Protein A column binds the Fc fragments and undigested IgG, allowing the Fab fragments to pass through the column. The Fc fragments from IgG4 and IgG1–2 were then eluted using IgG Elution Buffer (0.2 M Glycine/HCl, pH 3.0) and neutralized with Tris-HCl (pH 9.0). The purity of eluted fractions was analyzed by western blot, and the interaction of IgG4-Fc fragments with the Fc portions of IgG1–2 from Mf+ and Mf− was investigated using ELISA and western blot.

### Western blot analysis of eluted IgG Fc fractions

For the western blot analysis of the purity of the fractions, 1 μg of total IgG, digested IgG1–2 and IgG4 fractions, and the corresponding Fc fragments from Mf+ and Mf− plasma were loaded onto different lanes of a polyacrylamide gel (10–12%) and resolved by SDS-PAGE (100 V, 45–60 min). The resolved proteins were transferred onto nitrocellulose membranes (GE Healthcare, Freiburg, Germany) using a Bio-Rad Trans-Blot Turbo Transfer System (Bio-Rad, Germany). The membranes were then blocked with 3% BSA in Tris Buffered Saline (TBS) (Bio-Rad, Germany) for 1 h at room temperature prior incubation with the primary antibodies (mouse anti-human IgG4 Fc and IgG Fab (1:1000) (all from Thermo Scientific, Rockford, USA)) for 1.5 h at room temperature. The nitrocellulose membranes were then washed with TBS/0.05% Tween 20 before incubation for 1 h with alkaline phosphatase-conjugated goat anti-mouse IgG (1:500) (Bio-Rad Laboratories, USA). Immune complexes were finally detected with NBT (nitro blue tetrazolium) and BCIP (5-bromo-4-chloro-3-indolyl-phosphate, Bio-Rad Laboratories, USA).

### ELISA and western blot analysis of IgG4 Fc-IgG1-2 Fc interactions

To examine the IgG4 Fc-IgG1–2 Fc interactions, ELISA and specific western blot were used.

For ELISA, high-binding ELISA plates (Greiner Bio-One, Frickenhausen, Germany) were coated with 100 μl of purified IgG1–2 Fc fragments at the concentration of 1 μg/ml in PBS and incubated at 4 °C overnight. Control wells were incubated with PBS. The plates were washed 5 times with PBS/0.05% Tween 20 and blocked with PBS/1% BSA for 1 h at room temperature. After repeated washes, 100 μl/well of either IgG4-positive and IgG4-negative fractions of Mf+ and Mf− were added to the respective plates overnight at 4 °C. Wells were further washed and diluted biotin-conjugated mouse anti-human IgG4 (1:500) and IgG (1:20000) (all from Thermo Fisher Scientific, Rockford, USA) were added to the corresponding plates. After 2 h incubation at room temperature, plates were washed and revealed with 50 μl/well of Streptavidin-HRP for 45 min in the dark. After a final washing step, 50-μl/well TMB substrate solution was added to the plates and the reaction was stopped 15 min later with 25-μl/well 2N H_2_SO_4_ solution (Merck KGAA, Darmstadt, Germany). Optical density was measured at 450 nm using the SpectraMAX ELISA reader, and the results were expressed as arbitrary units (AU) relative to a unique plasma sample with the highest OD used as standard on all plates and arbitrarily set at 5 AU.

For western blotting, 1 μg of purified total IgG1–2, IgG1–2 Fc, and IgG1–2 Fab fragments from Mf+ and Mf− plasma were loaded onto 4–12% SDS-PAGE gels. After separation (100 V, 45–60 min) and transfer using a Bio-Rad Trans-Blot Turbo Transfer System (Bio-Rad, Germany), the nitrocellulose membranes (GE Healthcare, Freiburg, Germany) were blocked with 3% BSA in Tris Buffered Saline (TBS)) (Bio-Rad, Germany) for 1 h at room temperature before incubation with 1 μg/ml of IgG4 Fc and IgG4 Fab fragments from Mf+ and Mf− overnight at 4 °C. The membranes were thereafter washed and incubated with the primary antibodies (mouse anti-human IgG4 Fc and IgG4 Fab (1:1000) (all from Thermo Scientific, Rockford, USA)) for 1.5 h followed by the secondary antibody, alkaline phosphatase-conjugated goat anti-mouse IgG (1:500) (Bio-Rad Laboratories, USA), for 1 h. Fc-Fc interactions between IgG4 and IgG1–2 fragments were detected using NBT (nitro blue tetrazolium) and BCIP (5-bromo-4-chloro-3-indolyl-phosphate, Bio-Rad Laboratories, USA).

### Statistical analysis

To determine statistical differences between the different groups, the software PRISM 5.03 (GraphPad Software, Inc., La Jolla, USA) was used. Comparative analyzes among groups were conducted using a Kruskal-Wallis test with a Dunn’s nonparametric post hoc test (> 2 groups). In case of two groups, a Mann-Whitney *U* test was used. Significance was accepted when *p* < 0.05.

## Results

### Reduced affinity of IgG1 and IgG2 antibodies to C1q in Mf+ individuals

**Fig. 1:**
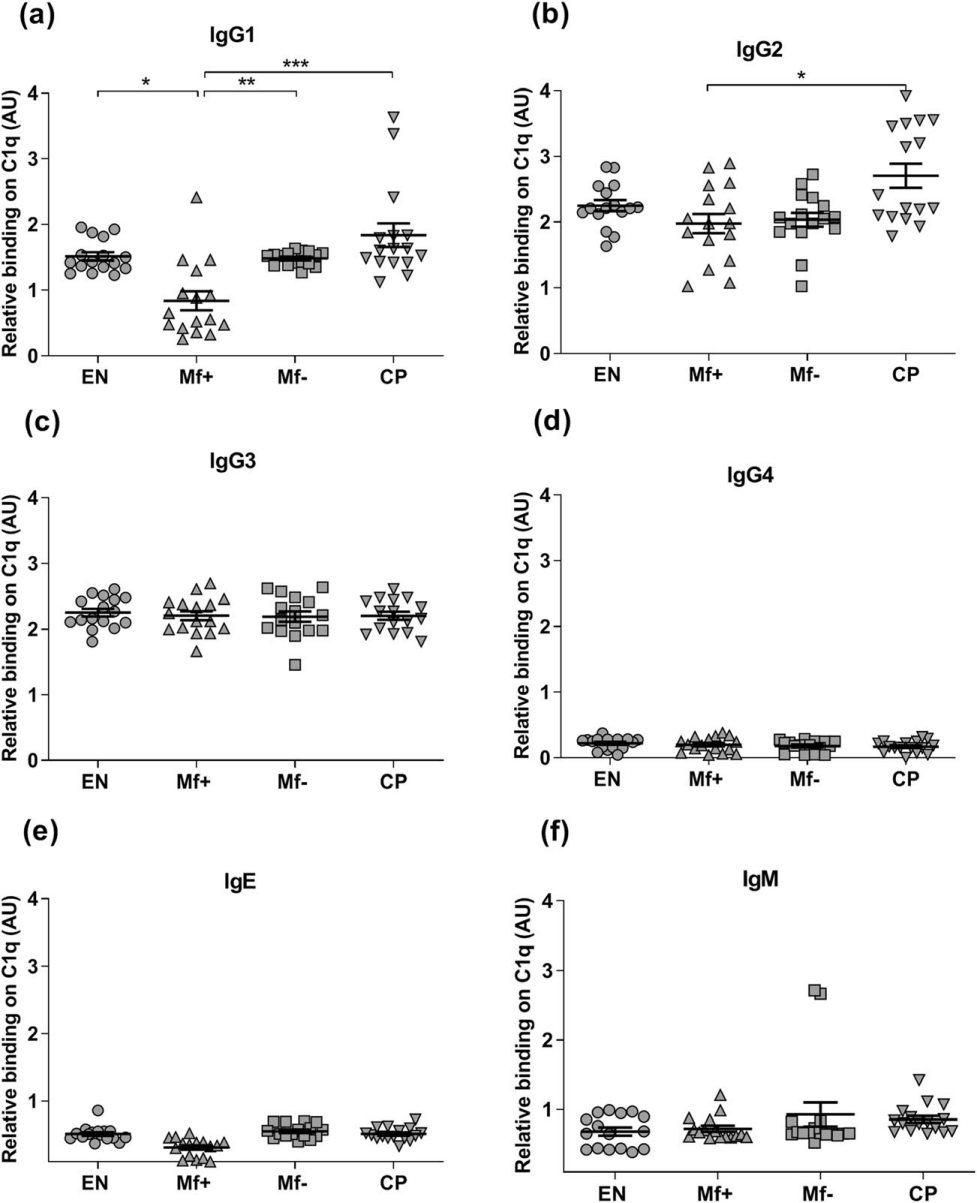
Binding capacity of IgG1 and IgG2 to C1q is reduced in Mf+ but not in CP patients. The ability of IgG1 (**a**), IgG2 (**b**), IgG3 (**c**), IgG4 (**d**), IgE (**e**), and IgM (**f**) from different plasma (*n* = 8) to bind plate-coated complement C1q was examined. Recombinant human C1q was coated at the concentration of 1 μg/ml. The plates were thereafter incubated with plasma samples from different individuals, and binding capacity of plasma immunoglobulins was revealed with biotin-conjugated anti-IgG1–4, IgE, and IgM antibodies. Plots represent means ± SEM of the relative binding capacity of each antibody expressed as arbitrary ELISA units (AU). Asterisks show statistical differences (Kruskal-Wallis one-way ANOVA followed by a Dunn’s multiple comparison test) between the groups. *P < 0.05; **P < 0.01; ***P < 0.001

To find out whether differences exist in the affinity of the antibody isotypes from the plasma of EN, Mf+, Mf−, and CP individuals to C1q, we investigated the capacity of each IgG subclass antibodies, IgE, and IgM in the plasma of individuals of the clinical groups to interact with C1q. While no difference was observed in C1q and IgG-C1q levels in EN, Mf+, Mf−, and CP (Online Resource 1), the results indicate that IgG1, IgG2, and IgE from the plasma differently fixed C1q (Fig. 10a, b, and e). In contrast, no difference was seen in the binding capacity of IgG3 and IgM (Fig. 1c, f). In addition, IgE displayed a low affinity to C1q. Nonetheless, the binding capacity of IgG1 from Mf+ individuals was significantly lower when compared to Mf−. In contrast, high affinity to C1q was detected for IgG1and IgG2 antibodies in CP patients. These results suggest a reduced capacity of pro-inflammatory antibodies from Mf+ individuals to interact with C1q.

### IgG4 depletion enhanced the affinity of IgG1 and IgG2 from Mf+ individuals to C1q

**Fig. 2:**
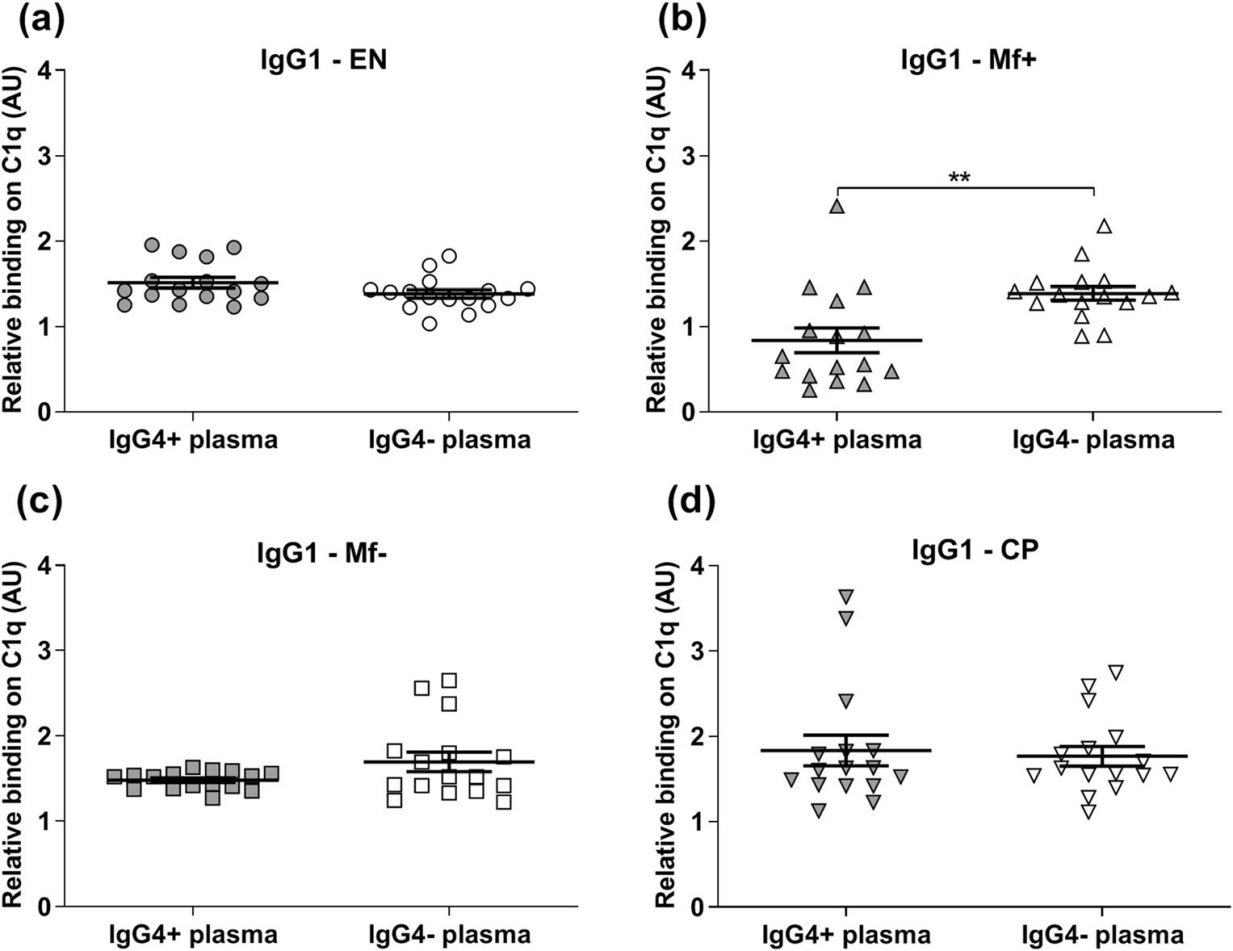
Removal of IgG4 from Mf+ plasma increased the binding capacity of IgG1 to complement C1q. The ability of IgG1 from EN (*n* = 8) (**a**), Mf+ (*n* = 8) (**b**), Mf− (*n* = 8) (**c**), and CP (*n* = 8) (**d**) to complement C1q in IgG4-positive plasma (gray plots) was tested by specific ELISA and compared with the corresponding IgG4-negative plasma (light plots) after removal of IgG4 antibodies. Results are expressed as arbitrary ELISA units (AU). Plots represent the relative binding capacity of IgG1 antibodies expressed as means ± SEM. Asterisks show statistical differences (Mann-Whitney test) between the two groups. *P < 0.05; **P < 0.01

**Fig. 3:**
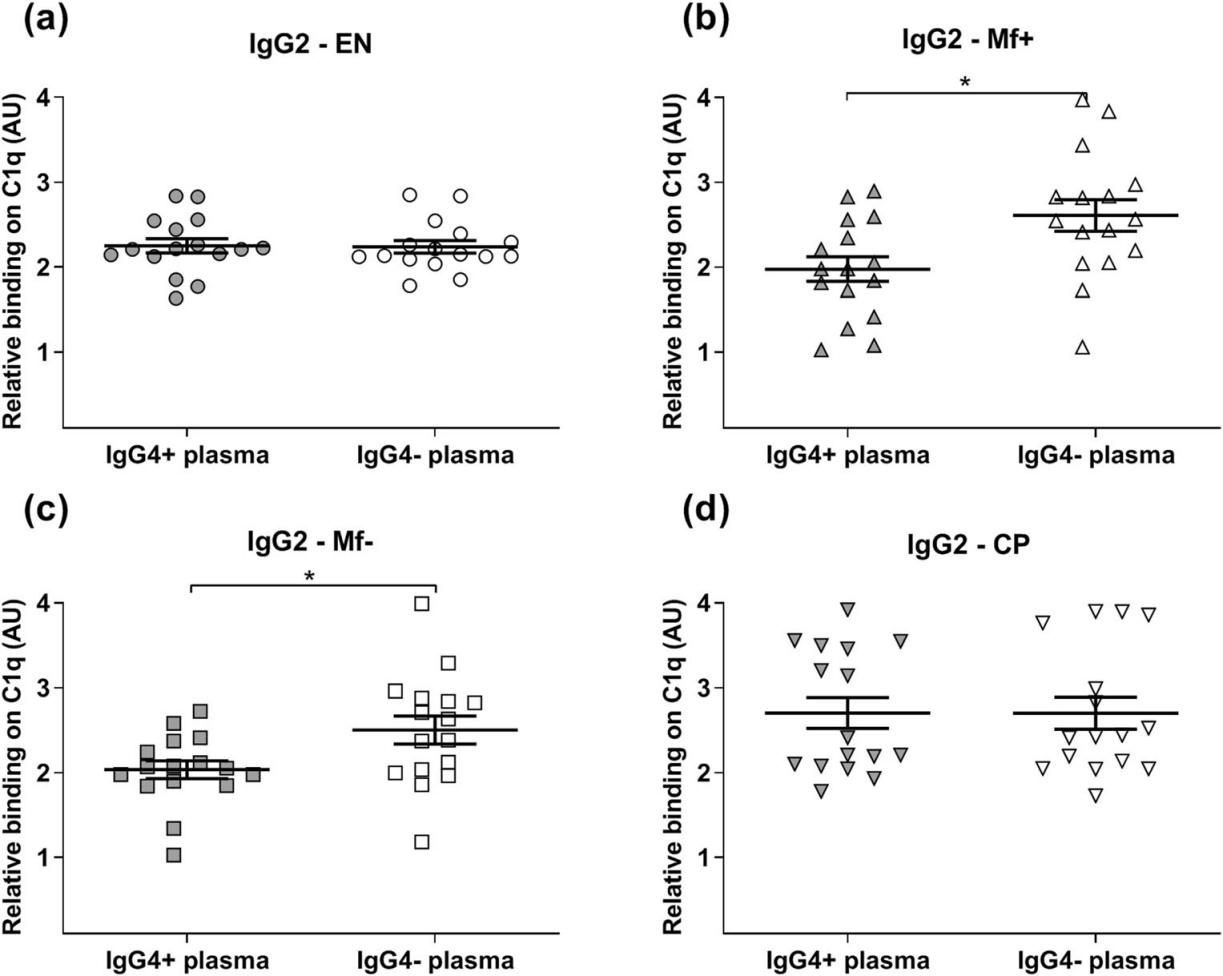
Removal of IgG4 from Mf+ and Mf− plasma increased the binding capacity of IgG2 to complement C1q. The ability of IgG2 from EN (*n* = 8) (**a**), Mf+ (*n* = 8) (**b**), Mf− (*n* = 8) (**c**), and CP (*n* = 8) (**d**) to complement C1q in IgG4-positive plasma (gray plots) was tested by specific ELISA and compared with the corresponding IgG4-negative plasma (light plots) after removal of IgG4 antibodies. Results are expressed as arbitrary ELISA units (AU). Plots represent the relative binding capacity of IgG2 antibodies expressed as means ± SEM. Asterisks show statistical differences (Mann-Whitney test) between the two groups. *P < 0.05; **P < 0.01

Since pro-inflammatory antibodies in Mf+ displayed a reduced capacity to interact with C1q and because previous data have suggested anti-inflammatory properties for IgG4, we investigated whether IgG4 antibodies are involved in the low affinity of IgG1and IgG2 to C1q in Mf+ individuals. Therefore, IgG4 was depleted from EN, Mf+, Mf−, and CP plasma. The levels of different immunoglobulin isotypes before and after IgG4 depletion were quantified (Online Resource 2) and the affinity of IgG1, IgG2, IgG3, IgM, and IgE to C1q in IgG4 depleted plasma was then analyzed. After IgG4 depletion, we observed a significant increase of the affinity of IgG1 and IgG2 from Mf+ plasma to interact with C1q (Figs. 2b and 3b). In contrast, no differences were seen in EN (Figs. 2a and 3a) and CP (Figs. 2d and 3d) plasma. In Mf− plasma, interestingly, whereas the depletion of IgG4 did not affect the affinity of IgG1 (Fig. 2c), IgG2’s affinity increased (Fig. 3c). These data suggest that IgG4 might be involved in the reduced affinity of IgG1 and IgG2 antibodies to complement C1q in Mf+.

**Fig. 4:**
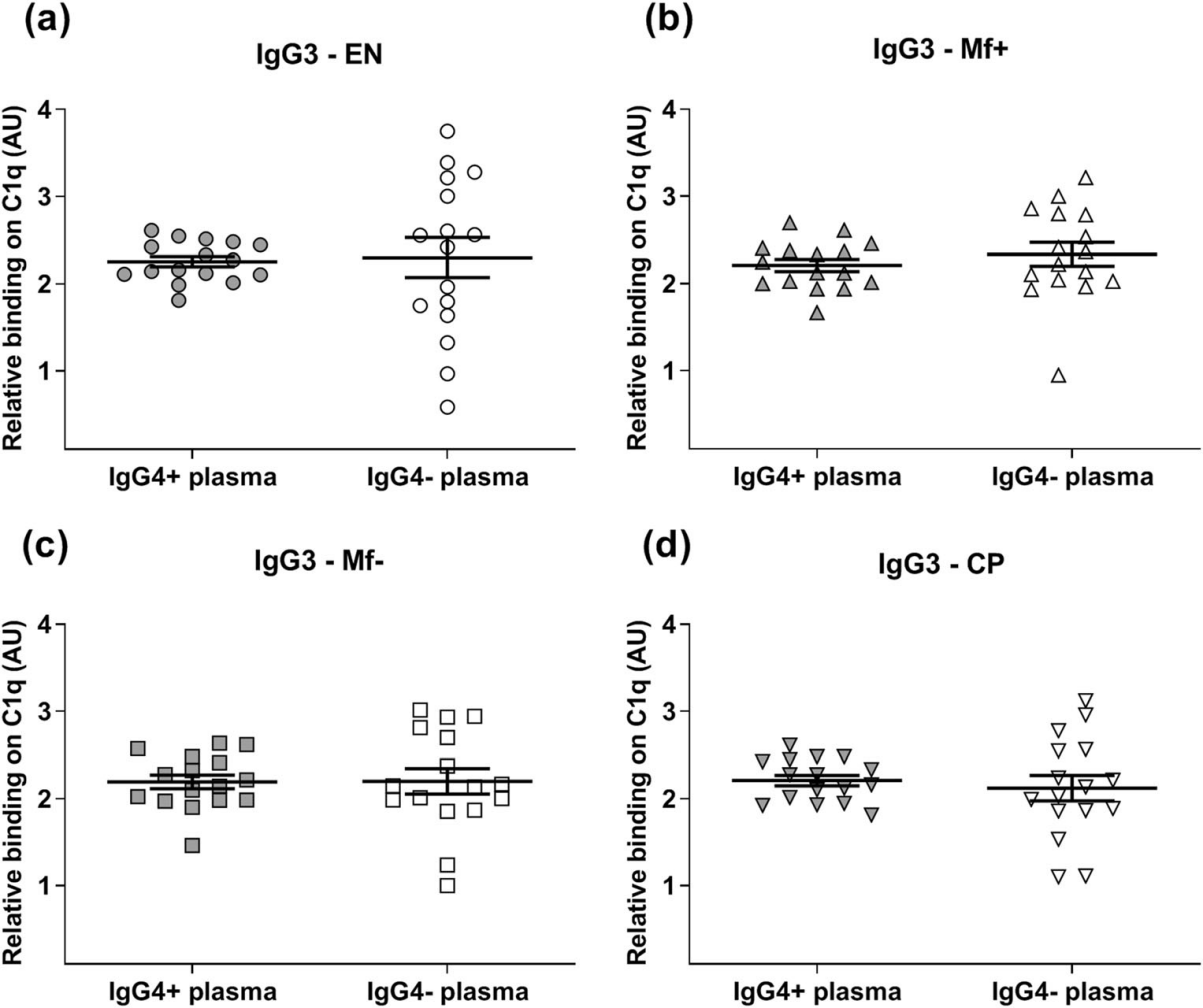
Depletion of IgG4 from EN, Mf+, Mf−, and CP plasma did not affect the binding capacity of IgG3 to complement C1q. The affinity of IgG3 from EN (*n* = 8) (**a**), Mf+ (*n* = 8) (**b**), Mf− (*n* = 8) (**c**), and CP (*n* = 8) (**d**) to complement C1q in IgG4-positive plasma (gray plots) was tested by specific ELISA and compared with the corresponding IgG4-negative plasma (light plots) after removal of IgG4 antibodies. Results are expressed as arbitrary ELISA units (AU). Plots represent the relative binding capacity of IgG3 antibodies expressed as means ± SEM

**Fig. 5:**
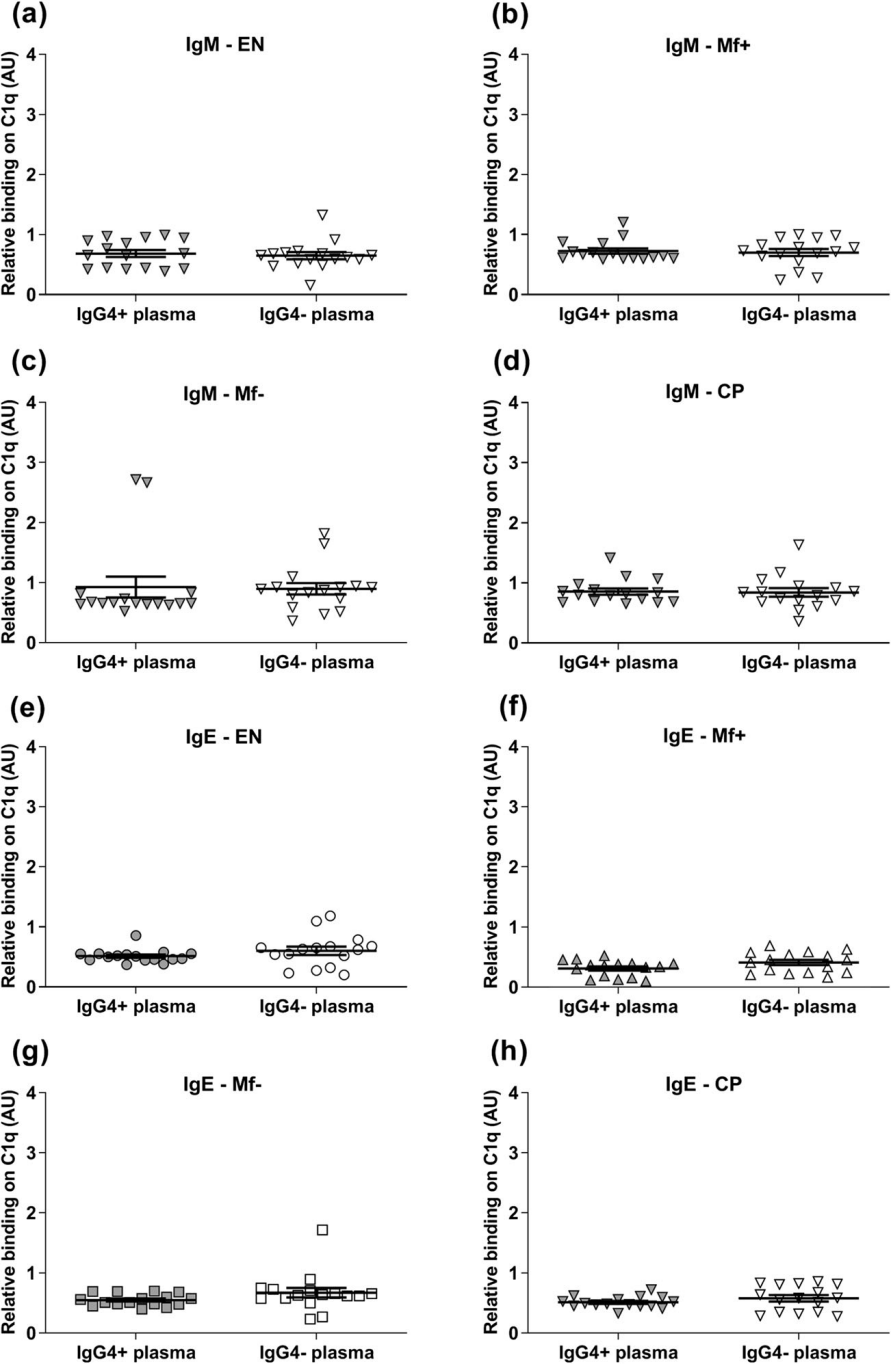
Depletion of IgG4 from EN, Mf+, Mf−, and CP plasma did not change the affinity of IgM and IgE to complement C1q. The affinity of IgM (**a**–**d**) and IgE (**e**–**h**) from EN (*n* = 8), Mf+ (*n* = 8), Mf− (*n* = 8), and CP (*n* = 8) to complement C1q in IgG4-positive plasma (gray plots) was tested by specific ELISA and compared with the corresponding IgG4-negative plasma (light plots) after removal of IgG4 antibodies. Results are expressed as arbitrary ELISA units (AU). Plots represent the relative binding capacity of IgM and IgE antibodies expressed as means ± SEM

Since the affinity of IgG1 and IgG2 to C1q was altered in the presence of IgG4 antibody, we further investigated whether these alterations also affect IgG3, IgM, and IgE. Interestingly, the results demonstrated that after IgG4 depletion from all groups, the affinity of IgG3 (Fig. 4a–d), IgM (Fig. 5a–d), and IgE (Fig. 5e–h) to C1q did not change. Also, the affinity of IgG remained particularly low in Mf+ plasma. Thus, IgG4 does not affect the affinity of IgG3, IgM, and IgE to complement C1q in Mf+ plasma.

### IgG4 interacted with IgG1–2 in a Fc-Fc-dependent mechanism

**Fig. 6:**
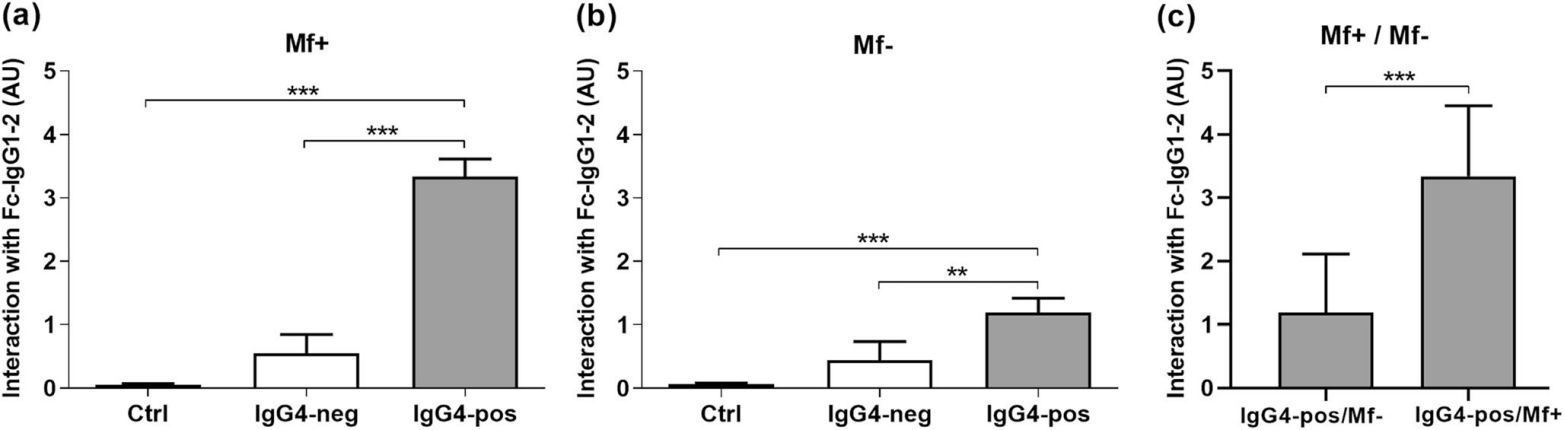
IgG4-positive but not IgG4-negative fractions from Mf+ and Mf− plasma interacted with the Fc fragment of IgG1–2. Interactions between coated IgG1–2 Fc and IgG4-negative (light plots) and IgG4-positive (gray plots) fractions from Mf+ (*n* = 8) (**a**, **c**) and Mf− (*n* = 8) (**b**, **c**) as well as the control (PBS/1% BSA) were analyzed by ELISA. After that, the ability of IgG4-positive fractions from Mf+ and Mf− to interact with IgG1–2 Fc was further compared (**c**). The results are expressed as arbitrary ELISA units (AU). Plots represent the relative interaction between the control (PBS/1% BSA), IgG4-positive and IgG4-negative fractions with Fc portion of IgG1–2 expressed as means ± SEM. Asterisks show statistical differences analyzed using Kruskal-Wallis one-way ANOVA followed by a Dunn’s multiple comparison test (**a**, **b**) and Mann-Whitney test (**c**) between the groups. **P < 0.01; ***P < 0.001

Next, we investigated the mechanism by which IgG4 may prevent interactions between inflammatory IgG1–2 antibodies and complement C1q. For this purpose, we explored whether the Fc fractions of IgG4 bind to the Fc fragment of IgG1 and IgG2 and prevented its interaction with C1q. Therefore, Fab and Fc fragments of IgG4 and IgG1–2 were generated and the purity of the fractions analyzed (Online Resource 3). We then investigated the interaction between IgG4-positive and IgG4-negative fractions of IgG4 and Fc portions of IgG1–2 from Mf+ and Mf−. We observed that binding did not occur with IgG4-negative fraction but rather with the positive fraction of Mf+ (Fig. 6a) and Mf− (Fig. 6b) compared to the control. In addition, the IgG4-positive/IgG1–2 Fc interaction was higher in Mf+ than Mf− (Fig. 6c).

**Fig. 7:**
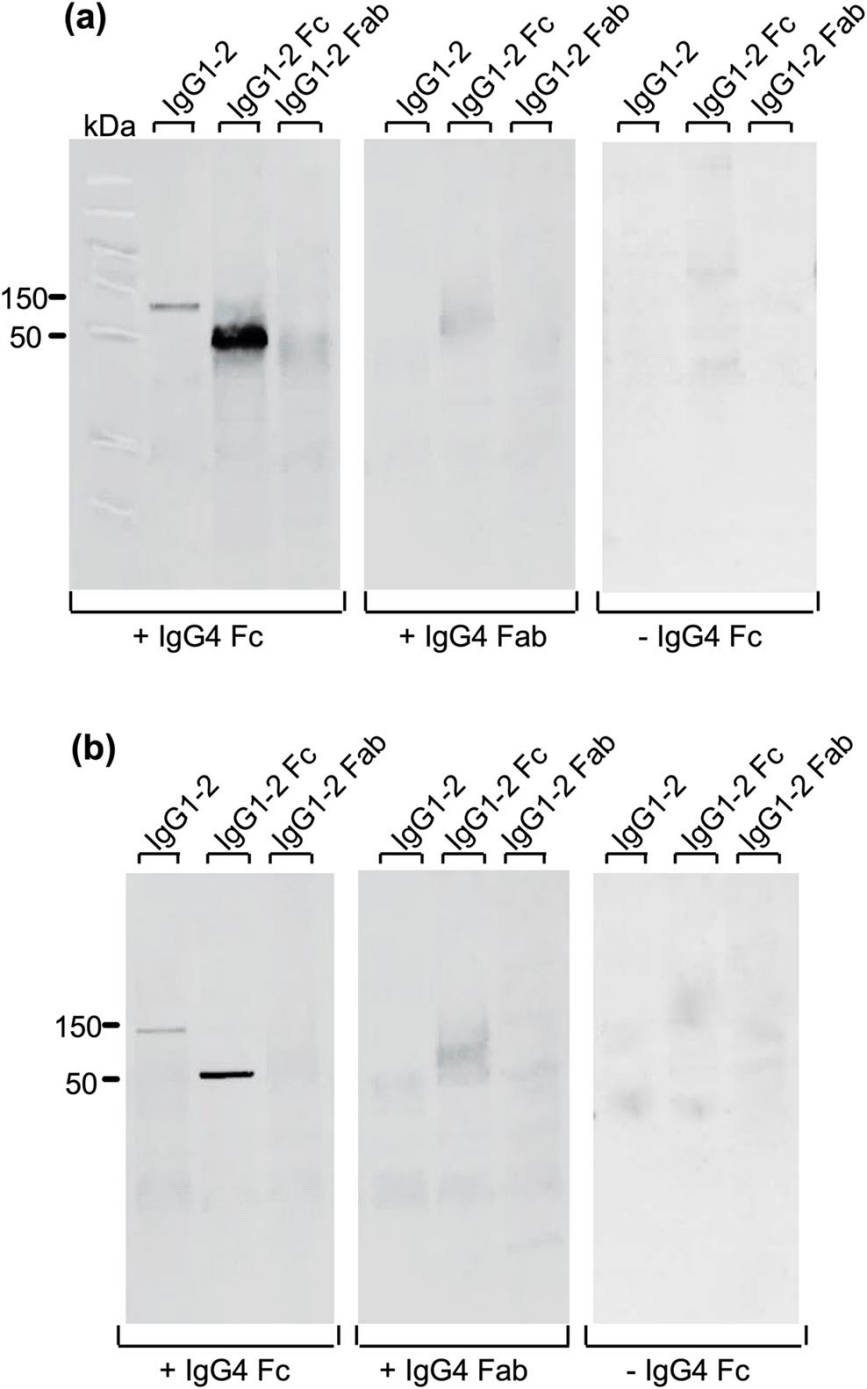
IgG4 Fc fragments from Mf+ and Mf- individuals interacted with the Fc fragments of IgG1–2. Fab and Fc fragments of IgG1–2 antibodies from Mf+ and Mf− were loaded on gels, transferred onto nitrocellulose membranes and incubated with either elution buffer (Tris-HCl neutralized) or with Fab and Fc portions of IgG4 from the corresponding group. Fc-Fc complexes characterized by the interactions between IgG4 Fab and Fc fragments of Mf+ (**a**) and Mf− (**b**) and the corresponding fragments of IgG1–2 from the same group were analyzed, and the size of the characteristic bands (150 kDa for total IgG1–2 and 50 kDa for IgG1–2 Fab and Fc fragments) was determined

Since IgG4-positive fraction interacted with the Fc part of IgG1 and IgG2, we further discriminated between the involvement of the Fc and Fab portions of IgG4 in this interaction by western blot. On Fig. 7a, we observed that the Fc part of IgG4 from Mf+ forms a complex with total IgG1–2 (150 kDa) and IgG1–2 Fc (50 kDa) but not with IgG1–2 Fab. In contrast, no complex with IgG4 Fab was observed. Similar results were obtained using IgG4 Fab and Fc fragments of Mf− (Fig. 7b). These results indicate that IgG4 Fc from Mf+ and Mf− interact with the Fc portion of IgG1–2, and this may be responsible for the reduced affinity of IgG1 and IgG2 antibodies to C1q observed above.

## Discussion

The complement system plays an essential role in the immune response against parasitic worms through interactions of C1q with antigen-bound immunoglobulins, primarily IgGs (IgG1–3), and IgM (Gahrton and Samson 2004), forming circulating immune complexes (CIC). These CICs are potent activators of the complement system and can profoundly affect the progression and outcome of lymphatic filariasis (Dixit et al. 2007; Kobayashi et al. 1997; Senbagavalli et al. 2011). In the present study, we examined the interaction between C1q and different antibody isotypes: IgG1–4, IgE, and IgM purified from EN, Mf+, Mf−, and CP individuals (Fig. 1), which also reflects the presence of C1q-binding immune complexes in the plasma as demonstrated elsewhere (Bratt and Ohlson 1988; Gazitt et al. 1985; Van Hoeyveld and Bossuyt 2000). Our data showed that pro-inflammatory antibody IgG1 from Mf+ individuals displayed a reduced affinity to C1q, the first element of the complement classical pathway when compared to Mf−, CP patients, and EN although no difference was observed in the levels of IgG-C1q among the groups. A similar trend was also seen for IgG2, even though a statistical significance was observed here only between Mf+ and CP patients. This finding together with the fact that C1q expression was similar in all samples indicates that molecular factors present in the plasma of Mf+ but not in EN, Mf−, and CP may inhibit the ability of IgG1 and 2 to bind to C1q.

Interestingly, our data on the expression of the protein C1q did not support previous observations by Yi Cai et al. in tuberculosis (TB) infections and recently confirmed by Lubbers et al. suggesting that C1q expression is increased in the peripheral blood of patients with active tuberculosis compared to healthy controls and individuals with latent TB infection (Cai et al. 2014; Lubbers et al. 2018). This apparent difference in the impact of clinical pathology on the expression of C1q is obviously due to core differences between the physiopathology of TB and LF. Indeed, since C1q is produced by phagocytotic cells such as macrophages and dendritic cells (Castellano et al. 2004; Loos et al. 1989), LF worms and microfilaria (> 1100 μm) that are too large to be phagocyted (Gray and Lawrence 2002) may have less impact on the C1q producing phagocytic cells compared to *Mycobacterium tuberculosis* which persistence mechanism consists in inhibiting the intracellular maturation of phagolysosome in these cells (Ufimtseva et al. 2019). Aging is another factor known to affect the expression of C1q (Kouser et al. 2015). The impact of age on the expression of C1q is however not relevant in the present study due to the relative homogeneity in the age of the donors used in the present study (Online Resource 1: Fig. S1b).

IgG4 antibodies count for approximatively 4% of total IgG in healthy individuals. Nonetheless, immune circumstances with chronic exposure to an antigen such as hyposensitization therapy with allergens (Aalberse et al. 2009) or helminth infections (Adjobimey and Hoerauf 2010) generally result in IgG4-dominated immune responses. Due to its molecular and immunological properties, IgG4 is often seen as a blocking antibody (Aalberse et al. 2009; Rispens et al. 2013; Rispens et al. 2009; van der Neut et al. 2007). Since, in LF models, IgG4 was shown to be predominant in Mf+ individuals compared to Mf−, CP, and EN (Prodjinotho et al. 2017), we hypothesized that IgG4 might be the factor that inhibited the affinity of IgG1 and IgG2 to C1q. To test this hypothesis, we depleted IgG4 from the plasma of Mf+ individuals and analyzed the ability of IgG1 and IgG2 in bulk Mf+ plasma (IgG4+) and IgG4-depleted plasma (IgG4−) to interact with C1q. Interestingly, both IgG1 and IgG2 presented a higher affinity to C1q in the IgG4-depleted context. This result suggests that even though IgG4 is incapable of binding to C1q, it modulated the affinity of both IgG1 and IgG2 to C1q. A similar conclusion was drawn by Van der Zee et al. who showed in a phospholipase A (PLA)-allergy model that IgG4-containing immune complexes were not capable of binding or to activate complement but inhibited the binding and complement activation by IgG1 anti-PLA antibodies (van der Zee et al. 1986). Due to technical limitations, we were not able to exclude a possible impact of a competition between IgG1 and IgG2. However, the outcome of such a competition is predictable since IgG1 is well known for having a higher affinity for C1q compared to IgG2 (Vidarsson et al. 2014). In addition, such a competition between IgG1 and IgG2 will potentially have no implication for our main finding that the presence of IgG4 modulates the affinity of both IgG1 and IgG2 to C1q.

Since IgG4 is known not to bind complement elements, it seems very unlikely that IgG4 antibodies inhibited IgG1/2 binding to C1q by interacting with C1q. In addition, our data clearly showed that IgG4 antibodies from all LF− clinical groups failed to interact with C1q. In their PLA model, van der Zee et al. suggested that IgG4 antibodies inhibited the binding of IgG1 to C1q by forming a complex with IgG1 (van der Zee et al. 1986). These observations were later confirmed by Rispens et al., who further demonstrated that IgG4 altered IgG1 activities including complement activation via Fc-Fc interactions (Rispens et al. 2013; Rispens et al. 2009). To investigate how IgG4-IgG1 or IgG4-IgG2 interactions may occur in our LF model, we generated Fab and Fc fragments of IgG4 and IgG1–2 using papain digestion and investigated how the different fragments interreacted using far-western blot analysis. Even if a residual affinity to IgG1–2 Fc fragments was detected in IgG4-depleted fractions, the values were not statistically significant, and our data suggest that total IgG4 and IgG4-Fc fragments interact with Fc fragments of IgG1–2. The ability of IgG4 to form Fc-Fc bounds with other antibodies is supported by the peerless flexibility in its CH3 domains (Davies et al. 2014; Davies and Sutton 2015).

Our data also clearly demonstrated that Fab fragments of IgG4 do not interact with IgG1–2 and that no interaction occurs between Fab portions of IgG1–2 and IgG4. Even though, we have not investigated whether IgG4 and IgG1–2 complexes present a reduced affinity to C1q, a recent study by Lilienthal et al. suggested that human IgG4 antibodies, as well as their murine counterpart IgG1, inhibit C1q binding by other IgG subclasses by preventing hexamer formation through steric interference (Lilienthal et al. 2018). This finding provides a very plausible explanation on how human IgG4 may *inhibit* C1q binding in our LF model and supports our conclusion that IgG4 antibodies inhibit the binding of IgG1 and IgG2 to C1q using a Fc-Fc-dependent mechanism.

Another interesting finding was the fact that in our model, IgG4 antibodies from Mf+ individuals presented a higher affinity to the Fc portions of IgG1–2 compared to IgG4 antibodies from Mf− and CP individuals. This finding extends our previous observation that functional differences regarding their affinity to FcγRs exist between IgG4 antibodies from LF patients of the different clinical groups (Prodjinotho et al. 2017) and suggest that a similar pattern might be applicable for their affinity to Fc fragments of other antibodies.

Our data also suggest that while IgG4 antibodies inhibit the ability of IgG1 and IgG2 to C1q, this blocking activity of IgG4 seems to not impact the affinity of IgG3 and IgM to the first element, the complement classical pathway. Indeed, our IgG4 depletion does not affect the affinity of these antibodies to C1q. This resistance to IgG4-mediated C1q-binding inhibition is certainly due to the very high affinity of these two isotypes to C1q. Indeed, it is well established that the globular domains of C1q preferentially recognize the Cγ2 domain of IgGs or the Cμ3 domain of IgM when these antibodies are complexed with antigen (Kaul and Loos 1997). In addition, among the IgG subclasses, C1q binds most efficiently and strongly to IgG3, followed by IgG1, but barely interacts with IgG2 while having no affinity to IgG4 (Flanagan and Rabbitts 1982). Thus, IgM and IgG3 are well known for having the highest affinity for C1q (Gadjeva et al. 2008). In line with our findings, a recent study using the murine surrogate of IgG4 (IgG1) showed that the higher the C1q-binding potential of an IgG subclass was, the less efficient was the inhibition by mouse IgG1, the murine surrogate of human IgG4 (Lilienthal et al. 2018).

In summary, our findings provide new evidence supporting the idea that IgG4 antibodies play a central role in immune evasion by helminth parasites and therefore represent a key target for the therapeutic control of the physiopathology of filarial infections.

## Electronic supplementary material

ESM 1 (PDF 244 kb)
