# Supplementary material for: IgG4 antibodies from patients with asymptomatic bancroftian filariasis inhibit the binding of IgG1 and IgG2 to C1q in a Fc-Fc-dependent mechanism
Source: Parasitol Res. 2019 Sep 4;118(10):2957–68. doi: 10.1007/s00436-019-06451-2 (PMC6754495; doi:10.1007/s00436-019-06451-2)
Supplement: Supplementary file 1 — (PDF 244 kb) [file 436_2019_6451_MOESM1_ESM.pdf]

Online Resources

Supplementary material 1

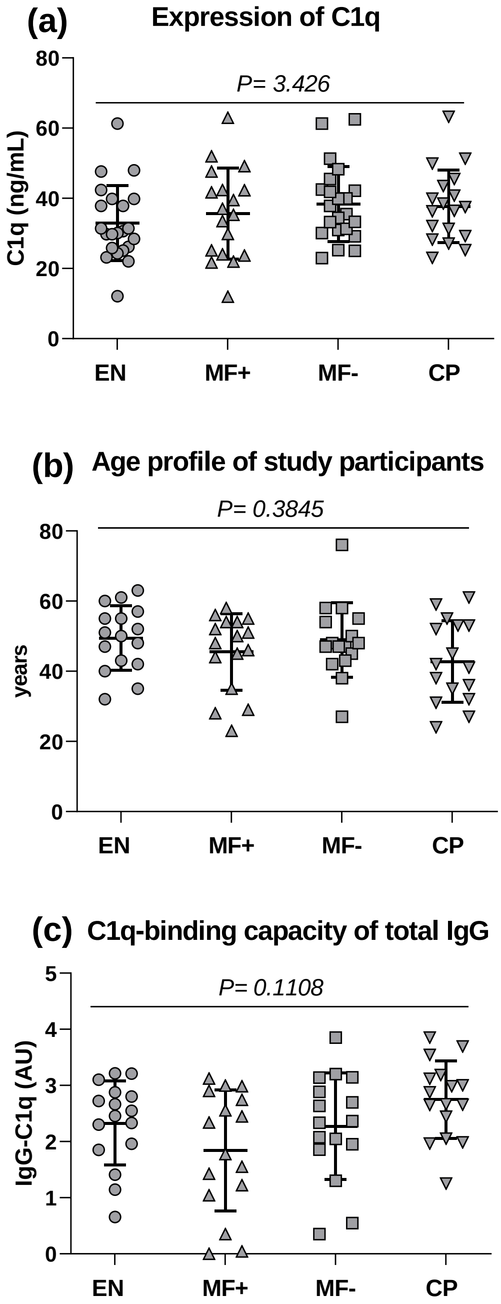

Supp Fig 1: C1q and IgG-C1q levels were similar in EN, Mf+, Mf- and CP. C1q (a) and IgG-C1q (c) levels in plasma samples from EN, Mf+, Mf- and CP were determined by ELISA and the age (in years) distribution of participants at recruitment (b) is shown. Plots represent means ± SEM of the levels of C1q and IgG-C1q and the age of participants in EN, Mf+, Mf- and CP groups. Asterisks show statistical differences (Kruskal-Wallis one-way ANOVA followed by a Dunn's multiple comparison test) between the groups. *P < 0.05; **P < 0.01; ***P < 0.001.

Supplementary material 2

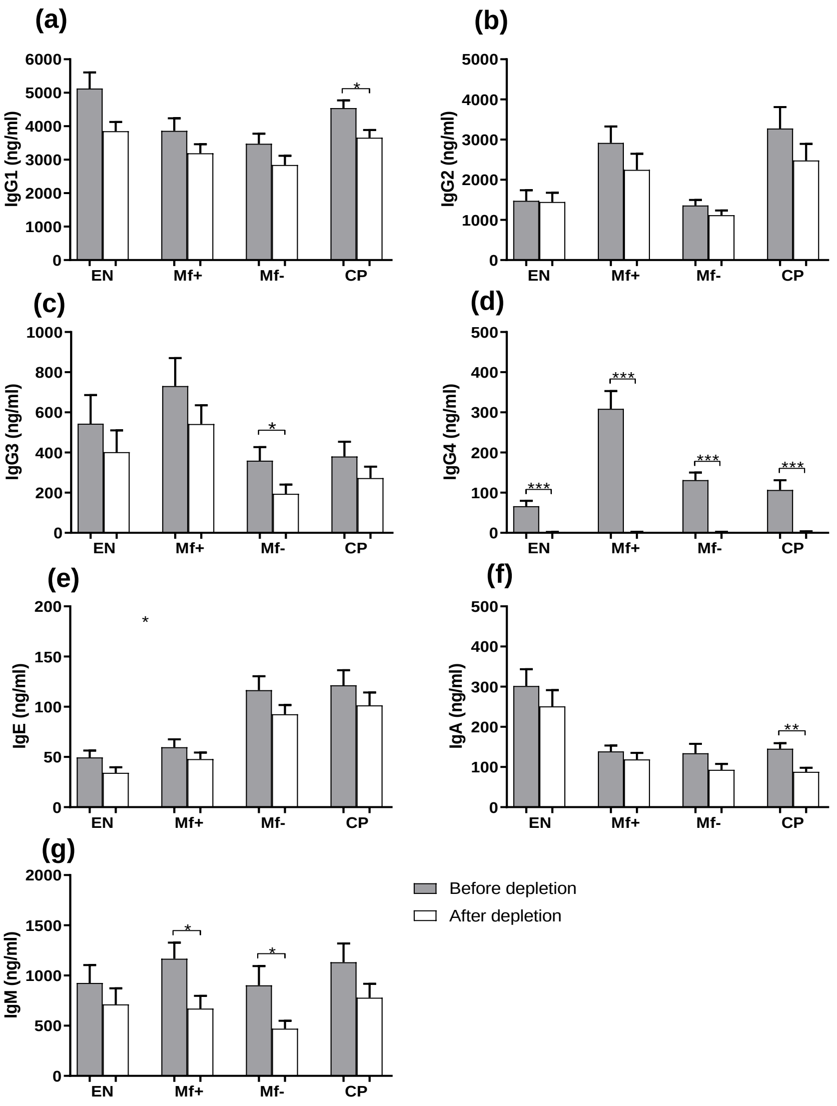

Supp Fig 2: Immunoglobulin isotype quantification in EN, Mf+, Mf- and CP plasma before and after IgG4 depletion. Diluted plasma from EN (n=8) and LF infected Mf+ (n=8), Mf- (n=8) and CP (n=8) individuals were analyzed for the levels of IgG1-4 (a-d), IgE (e), IgA (f) and IgM (g) before (grey bars) and after IgG4 depletion (light bars) using Luminex-based immunoassay. Bars depict the antibody levels expressed as mean ± SEM. Asterisks show statistical differences (Mann-Whitney test) between the two groups. *P < 0.05; **P < 0.01; ***P < 0.001.

Supplementary material 3

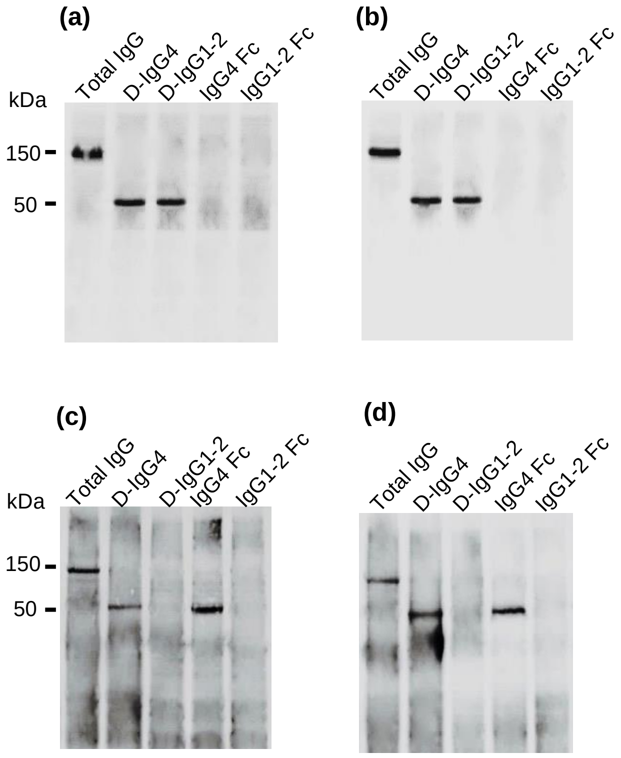

Supp Fig 3: **Purity of generated IgG4, IgG1-2 Fab and Fc fragments from Mf+ and Mf- plasma.** Total IgG, digested IgG4 (D-IgG4) and IgG1-2 (D-IgG1-2) and the Fc fragments of IgG4 and IgG1-2 antibodies from Mf+ (a, c) and Mf- (b, d) were loaded on gels, transferred onto nitrocellulose membranes. Then the presence of IgG (150 kDa), Fab (50 kDa) and Fc (50 kDa) characteristic bands on membranes was analyzed using mouse anti-human IgG Fab (a, b) and IgG4 Fc (c, d) antibodies and revealed with goat anti-mouse IgG-AP.
